# A Sensible Idea

**Published:** 1890-11

**Authors:** 


A SENSIBLE IDEA.—A new innovation at funerals here recently is a silk skull cap, to be worn by the minister in charge and the bearers at the grave, also by the male members of the family, the caps are put on in the carriage and the ordinary hats left there, the caps to be worn all the time at the grave. It will prevent many colds.—*American Druggist.*

